# Supplementary material for: Flavanol Polymerization Is a Superior Predictor of α-Glucosidase Inhibitory Activity Compared to Flavanol or Total Polyphenol Concentrations in Cocoas Prepared by Variations in Controlled Fermentation and Roasting of the Same Raw Cocoa Beans
Source: Antioxidants (Basel). 2019 Dec 11;8(12):635. doi: 10.3390/antiox8120635 (PMC6943598; doi:10.3390/antiox8120635)
Supplement: Supplementary file 1 [file antioxidants-08-00635-s001.pdf]

antioxidants

MDPI

Supporting Information

Flavanol polymerization is a superior predictor of α-glucosidase inhibitory activity compared to flavanol or total polyphenol concentrations in cocoas prepared by variations in controlled fermentation and roasting of the same raw cocoa beans

Kathryn C. Racine[1], Brian D. Wiersema[1], Laura E. Griffin[2], Lauren A. Essenmacher[1], Andrew H. Lee[1], Helene Hopfer[3], Joshua D. Lambert[3], Amanda C. Stewart[1], Andrew P. Neilson[2*]

[1]Department of Food Science and Technology, Virginia Polytechnic Institute and State University, Blacksburg, VA 24060
[2]Plants for Human Health Institute, Department of Food, Bioprocessing and Nutrition Science, North Carolina Research Campus, 600 Laureate Way, Kannapolis, NC 28081
[3]Department of Food Science, Pennsylvania State University, University Park, PA 16801

Corresponding Author:
Dr. Andrew P. Neilson, Plants for Human Health Institute, Department of Food, Bioprocessing and Nutrition Sciences, North Carolina State University, 600 Laureate Way, Kannapolis, NC 28081; Phone: 704-250-5495; Fax: 704-250-5409; Email: aneilso@ncsu.edu

---

Materials and Methods

Polyphenol extraction and quantification: Randomly selected whole cocoa beans (30 g) were frozen with liquid nitrogen and ground into powder. Cocoa powders (40 g, from pressing or from ground beans) were mixed with 150 mL hexane and sonicated (10 min, 22°C), centrifuged for (5 min, 5000 × *g)*, supernatant discarded, and then repeated. Once defatted, the powder was dried at room temperature. Once dry, defatted powder was mixed with 150 mL of extraction solution (70:28:2 acetone, water, acetic acid v/v/v), sonicated (10 min, 22°C), and centrifuged for (5 min, 5000 × *g*). The supernatant was collected and this procedure was repeated three more times for a total volume of 600 mL. All collected supernatant was pooled and placed under vacuum on a rotary evaporator at 40°C until all acetone evaporated. The resulting cocoa extract was frozen at −80°C, freeze dried, and the final extract was weighed to calculate extract yield. The freeze-dried extract was stored at −80°C until further analysis.

Folin-Ciocalteu colorimetric assay: Cocoa extracts (n=3) were diluted with 40% EtOH to a final concentration of 0.2 mg/mL. In a 96-well plate, each solution (5 µL) was mixed with MQ water (45 µL) and 0.2 N Folin-Ciocalteu reagent (125 µL). 100 µL sodium carbonate solution (7.5% v/v) was added to the samples and mixed. The plate was incubated for 2 h at room temperature and the absorbance read at 765 nm. Samples were compared to gallic acid standard curves (n=2). Total polyphenol concentrations were expressed as mg Gallic Acid Equivalents (GAE)/g cocoa bean.

4-dimethylaminocinnamaldehyde (DMAC) colorimetric assay: DMAC solution was prepared by combining 3.0 mL stock HCl with 27 mL EtOH and chilling at 4°C for 15 min, 0.03 g DMAC was added to the solution and mixed well. Cocoa extracts were diluted with EtOH to a final concentration of 100 ppm. Standard curve (n=2) was prepared by diluting procyanidin B2 with 1:1 EtOH:water to concentrations of 1, 10 50, and 100 ppm. In a 96-well plate, each diluted cocoa extract, PCB2 standard (1, 10, 50, 100 ppm), and EtOH blank (50 µL) was mixed with 250 µL of DMAC solution. Absorbance was read at 640 nm.
Thiolysis: Cocoa extracts were diluted with MeOH to 0.5 mg/mL and then mixed (50 µL) with 50 µL HCl (3.3%, water) and 100 µL benzyl mercaptan (5%, MeOH). Samples were placed in a 90°C water bath for 5 min and then cooled on ice for 5 min. Unthiolyzed controls were prepared with cocoa extract and MeOH without heating in the water bath. Each thiolyzed sample (100 µL) was combined with 900 µL of 0.1% formic acid in water and 0.1% formic acid in ACN (95:5 v/v). Samples were analyzed on a Waters Acquity H-Class separations module with an Acquity UPLC HSS T3 column (2.1 mm × 100 mm, 1.8 µm) at 40°C. Binary gradient elution was performed using 0.1% formic acid in water (Phase A) and 0.1% formic acid in ACN (Phase B). Solvent flow rate was 0.6 mL/min and the linear gradient elution was as followed: 95% A (0-0.5 min), 65% A (6.5 min), 20% A (7.5-8.6 min), 95% A (8.7-10.5). (−)-electrospray ionization (ESI) together with tandem mass spectrometry (MS/MS) was used to analyze UPLC effluent on a Waters Acquity triple quadrupole (TQD) MS. (−) mode electrospray ionization (ESI) was performed with capillary, cone, and extractor voltages of –4.24 kV, 30.0 V, and 3.0 V respectively. Source temperature was 150°C and desolvation temperature was 400°C/ Cone gas flowed at a rate of 75 L/h and desolvation gas at 900 L/h. Argon (0.25 mL/min) was used as the collision gas in MS/MS. Multi-reaction monitoring (MRM) with a mass span of 0.2 Da was performed on parent ions and collision-induced dissociation (CID) on daughter ions. Inter-channel delays and interscan time was 1.0 s each. Additional calculations were done to account for the native monomers and were reported as DP of total flavanols. mDP oligomers and polymers and mDP of total flavanols were calculated as follows:

$$mDP\ (O+P) = \frac{net\ number\ of\ monomers + net\ number\ of\ thiolytic\ derivatives}{net\ number\ of\ monomers}$$

$$mDP\ (total\ flavanols) = \frac{total\ monomers + net\ number\ of\ thiolytic\ derivatives}{total\ monomers}$$

HILIC UPLC-MS/MS: A Waters Acquity H-class separation module equipped with an Acquity Torus DIOL column (2.1 mm × 100 mm, 1.7 µL, 45°C) and Torus DIOL VanGuard Pre-column (2.1 mm × 5 mm, 1.7µL) was used to perform the analysis. Binary gradient elution was performed with 2% acetic acid in acetonitrile (phase A) and 3% water and 2% acetic acid in methanol (phase

B). Solvent flow rate was 0.8 mL/min and the linear gradient elution was carried out as followed: 100% A (0 min), 55% A (5.7 min), 5% A (6.0 min), 100% A (6.7-9.0 min). (−)- mode ESI coupled to tandem mass spectrometry (MS/MS) on a Waters Acquity triple quadrupole (TQD) MS was used to analyze the UPLC-eluent. Ammonium formate (0.04 M in water, 5 µLmin) was added to the eluent flow stream post-column to enhance ionization of the high molecular weight compounds. Ionization settings were as follows (−) mode, capillary voltage: −4.5 kV, cone voltage: 60.0 V, extractor voltage: 1.0 V, source temperature: 150°C, and desolvation temperature: 500°C. N_2 was used for cone and desolvation gasses with flow rates of 50 and 1000 L/h respectively. For MS/MS, Ar was used as a collision gas with 0.1 mL/min flow rate. Parent ions and signature daughter ions followed by collision-induced dissociation (CID) were subjected to multi-reaction monitoring (MRM) with a mass span of 0.2 Da and 1.0 sec of inter-channel delays and inter-scan times. A calibration curve for standards DP 1-9 were prepared and analyzed with dilutions ranging from 6.93×10^{-7} – 0.091 mg/mL. MRM settings for each compound are listed in Table S1. MassLynx software (version 4.1, Waters) was used to acquire data.

Table S1. *MS/MS settings for MRM detection of monomer-decamer flavanols*

**Compound**	**t_R[a] (min)**	**MW ($g\ mol^{-1}$)**	**$[M – H]^{-b}$ (m/z)**	**Daughter Ion (m/z)**
Monomer	0.61	290.27	289.03	245.06
Epigallocatechin	0.74	458.37	305.04	124.98
Dimer	2.03	578.52	577.14	425.10
Trimer	3.05	866.77	865.22	287.07
Tetramer	3.73	1155.02	576.40	125.02
Pentamer	4.26	1443.28	720.41	125.02
Hexamer	4.66	1731.53	864.52	125.02
Heptamer	5.00	2017.81	1008.40	125.17
Octamer	5.28	2308.03	1152.58	125.17
Nonamer	5.53	2596.54	864.12	125.17
Decamer	5.75	2884.54	960.18	125.17

[a]retention time

[b]All MRMs used singly-charge parent ions except for pentamer, hexamer, heptamer, and octamer, which are double-charged ($[M – 2H]^{2-}$), and nonmaer and decamer, which are triple-charged ($[M – 3H]^{3-}$)

Preliminary melanoidin dialysis: A polyphenol-rich cocoa extract was prepared by our standard method of defatting 2X with hexane and extracting a minimum of 3X (or until the supernatant has no color) using an extraction solvent of acetone, water, and acetic acid (70:28:2 v/v/v). Pooled extracts were dried by rotary evaporation to remove acetone and then freeze dried to remove water. The dialysis method proposed by Sacchetti et al [1] was followed with modifications. Cocoa extract was re-dissolved in extraction solvent to a final concentration of 40 mg/mL. Dialysis was preformed in triplicate using acidified MeOH:water (60:40 v/v, 0.1% formic acid). For each replicate, 10 mL of 40 mg/mL cocoa extract was placed inside approximately 30 cm of presoaked dialysis tubing (3.5-5.0 kDa MW cutoff, Spectrum Spectra/Por Biotech-Grade RC Dialysis, Fisher) and clipped closed. The tubing was submerged in 1L of dialysis solvent and stirred at 4°C for 24 h. After 24 h, the remaining cocoa extract constituents within the dialysis tubing was transferred into a new presoaked tube (8.0-10.0 kDa MW cutoff) and clipped closed. This tubing was placed into a new 1 L beaker of fresh dialysis solvent and stirred at 4°C for 24 h. This sequence was repeated with MW cutoff of 20 kDa and 50 kDa. Dialysis beakers were continually sparged with nitrogen throughout the 24 h period. Samples of the acidified MeOH:water were taken after every 24 h period and the cocoa extract remaining within the dialysis tubing was collected upon completion, and all samples were frozen at −80°C until analysis.

To selectively quantify MRP, each dialysate (<3.5-5 kDa, <8-10 kDa, <20 kDa, <50 kDa) was diluted 10-fold with 0.05 H_2SO_4. The non-dialyzable cocoa extract (>50 kDa) was diluted 10-fold with 0.5 M H_2SO_4 and then further diluted with 0.05 M H_2SO_4 until the solution was colorless. The starting cocoa extract (40 mg/mL) was diluted 10-fold with 0.5 M H_2SO_4 and then further diluted with 0.05 M H_2SO_4 until the solution was colorless. A

standard curve was prepared with quinine sulfate dissolved in 0.05 M H_2SO_4 (100-0.1 ppm). Each diluted dialysate, diluted non-dialyzable cocoa extract, diluted starting cocoa extract, and standard was transferred (300 µL) into a UV-Star 96-well plate. The absorbance was read at 280, 360, and 420 nm and early, intermediate, and late MRP were reported as absolute absorbance values in Table S2.

Results

<u>Preliminary melanoidins dialysis:</u> Early, intermediate, and late MRP of cocoa extract were identified from LMW (<3.5-5 kDa, <8-10 kDa, <20 kDa, <50 kDa) and HMW (>50 kDa) fractions. The majority of these MRP were eluted from the 3.5-5 and 8-10 kDa membranes, followed by minimal compounds in 20 kDa, and increasing amounts of early, intermediate and late MRP eluted from the 50 kDa membrane. Additionally, the non-dialyzable HMW cocoa extract (>50 kDa) had significant levels of compounds detected, suggesting that a large quantity of MRP compounds within roasted cocoa are extremely large and warrant further investigation into their identification and quantification.

Table S2. *Preliminary melanoidin identification*

**Fraction**	**Early MRP[a] (280 nm)**	**Intermediate MRP[a] (360 nm)**	**Late MRP[a] (420 nm)**
<3.5-5 kDa	2.295	0.11	0.052
<8-10 kDa	0.162	0.046	0.038
<20 kDa	0.106	0.043	0.037
<50 kDa	0.401	0.064	0.05
>50 kDa	2.922	0.455	0.327
Starting extract	3.711	0.287	0.152

[a]Values are reported as absolute absorbance

Figures and Tables

Table S3. *Specifications for cocoa liquors and cakes of each treatment*

**Treatment**	**Liquor (%)**		**Cake (%)**		**Liquor Particle Size (µm)[a]**			
	**Fat**	**Moisture**	**Fat**	**Moisture**	**99% through**	**95% through**	**75% through**	**50% through**
UF/UR	55.96	1.43	9.15	5.23	88.00	52.00	18.50	11.00
UF/CR	58.10	1.36	8.70	3.43	124.50	74.00	18.50	11.00
UF/HR	56.78	0.73	15.36	2.01	209.30	114.10	20.17	10.09
CF/UR	58.20	2.37	10.97	4.38	191.90	104.60	23.99	12.00
CF/CR	58.04	1.37	9.32	2.82	191.90	104.60	22.00	11.00
HF/UR	56.03	1.98	9.17	4.80	67.86	47.98	20.17	11.00
HF/HR	58.06	1.09	7.50	3.00	148.00	74.00	16.96	10.09

[a]Headings indicate the % of cocoa liquor passing through 10 mm mesh screen

Table S4. *Compositional data analysis as determined by 2-way ANOVA for roasting and fermentation effect using type III sums of squares to account for unbalanced data. Normality was checked for each variable visually and with the Shapiro-Wilks test, and if needed, transformed prior to running the 2-way ANOVA. Significance determined by *p<0.05, **p<0,01, ***p<0.001.*

Measure		Roasting	Fermentation	Roasting:Fermentation	Residuals
Folin-Ciocalteu	*Df*	2	2.00E+00	2.00E+00	14
	SS	596.81	861.23	146.9	444.77
	F	9.3929	13.5544	2.312	
	p	0.0025888	0.0005313	0.1356369	
DMAC	*Df*	2	2.00E+00	2.00E+00	14
	SS	63.475	103.37	18.189	26.502
	F	16.7659	27.3033	4.8042	
	p	0.0001923	1.47E-05	0.0257892	
Thiolysis (Including Monomers)	*Df*	2	2.00E+00	2.00E+00	28
	SS	0.50	1.85	1.92	1.79
	F	3.9047	14.4968	15.0068	
	p	0.03193	4.77E-05	3.72E-05	
Thiolysis (Oligomers/ Polymers)	*Df*	2	2.00E+00	2.00E+00	28
	SS	1.417	36.213	2.297	13.289
	F	1.4929	38.1504	2.4202	
	p	0.2421	1.01E-08	0.1073	
Total Procyanidins[a]	*Df*	2	2.00E+00	2.00E+00	14
	SS	1.64191	1.84269	0.01062	0.01459
	F	787.7787	884.1134	5.0978	
	p	4.11E-15	1.85E-15	2.17E-02	
Monomers[b]	*Df*	2	2.00E+00	2.00E+00	14
	SS	0.015565	0.066195	0.009732	0.001271
	F	85.712	364.51	53.591	
	p	1.40E-08	8.43E-13	2.75E-07	
Dimers	*Df*	2	2.00E+00	2.00E+00	14
	SS	0.1778	0.263889	0.010809	0.008467
	F	147	218.1759	8.9365	
	p	4.01E-10	2.81E-11	0.003155	
Trimers	*Df*	2	2.00E+00	2.00E+00	14
	SS	1.78642	2.4954	0.27048	0.03787
	F	330.236	461.298	50.001	
	p	1.66E-12	1.67E-13	4.21E-07	
Tetramer	*Df*	2	2.00E+00	2.00E+00	14
	SS	0.68862	0.91209	0.11974	0.0134
	F	359.728	476.464	62.552	
	p	9.23E-13	1.33E-13	1.05E-07	
Pentamer[a]	*Df*	2	2.00E+00	2.00E+00	14

	SS	1.64191	1.84269	0.01062	0.01459
	F	787.7787	884.1134	5.0978	
	p	4.11E-15	1.85E-15	2.17E-02	
Hexamer	*Df*	2	2.00E+00	2.00E+00	14
	SS	2.41207	2.28987	0.43301	0.04927
	F	342.716	325.353	61.524	
	p	1.29E-12	1.84E-12	1.16E-07	
Heptamer[a]	*Df*	2	2.00E+00	2.00E+00	14
	SS	1.91242	2.30246	0.01559	0.0418
	F	320.2337	385.5464	2.6099	
	p	2.05E-12	5.73E-13	1.09E-01	
Octamer	*Df*	2	2.00E+00	2.00E+00	14
	SS	9.5584	7.1018	1.6975	0.1247
	F	536.703	398.762	95.313	
	p	5.86E-14	4.55E-13	7.02E-09	
Nonamer	*Df*	2	2.00E+00	2.00E+00	14
	SS	11.2358	7.8036	1.8694	0.3498
	F	224.84	156.16	37.41	
	p	2.29E-11	2.68E-10	2.42E-06	
Decamer[a]	*Df*	2	2.00E+00	2.00E+00	14
	SS	2.29234	2.21647	0.00711	0.02807
	F	571.65	552.7354	1.7734	
	p	3.79E-14	4.78E-14	2.06E-01	
Early MRP <8-10 kDa	*Df*	2	2.00E+00	2.00E+00	14
	SS	0.02807	0.33149	0.27022	0.5754
	F	0.3414	4.0327	3.2874	
	p	0.7165	0.04139	0.06754	
Early MRP >8-10 kDa	*Df*	2	2.00E+00	2.00E+00	14
	SS	0.052822	0.071489	0.04712	0.074933
	F	4.9345	6.6782	4.4018	
	p	0.023882	0.009193	0.032876	
Early MRP Unfractionated CE	*Df*	2	2.00E+00	2.00E+00	14
	SS	0.03889	0.1482	0.065953	0.071311
	F	3.8174	14.5475	6.474	
	p	0.0475132	0.0003819	0.0102145	
Intermediate MRP <8-10 kDa	*Df*	2	2.00E+00	2.00E+00	14
	SS	0.41376	0.0338	0.01348	0.0976
	F	29.6751	2.4242	0.9665	
	p	9.23E-06	0.1247	0.4044	
Intermediate MRP >8-10 kDa[a]	*Df*	2	2.00E+00	2.00E+00	14
	SS	0.13144	0.39248	0.20491	0.4509
	F	2.0405	6.0931	3.1812	
	p	0.16685	0.01248	0.07263	

Intermediate MRP Unfractionated CE	*Df*	2	2.00E+00	2.00E+00	14
	SS	0.74964	0.05581	0.47902	0.01601
	F	327.817	24.405	209.473	
	p	1.746E-12	0.00002733	3.697E-11	
Late MRP <8-10 kDa	*Df*	2	2.00E+00	2.00E+00	14
	SS	0.133622	0.037489	0.005253	0.064667
	F	14.4643	4.0581	0.5687	
	p	0.0003924	0.0407314	0.5788331	
Late MRP >8-10 kDa[a]	*Df*	2	2.00E+00	2.00E+00	14
	SS	0.05766	0.93845	0.4373	0.69484
	F	0.5809	9.4542	4.4055	
	p	0.572338	0.002522	0.032801	
Late MRP Unfractionated CE	*Df*	2	2.00E+00	2.00E+00	14
	SS	2.57511	0.15782	1.6337	0.093
	F	193.593	11.879	122.962	
	p	6.302E-11	0.0009636	1.315E-09	

[a]Data normalized through Log transformation

[b]Data normalized through Box-Cox transformation

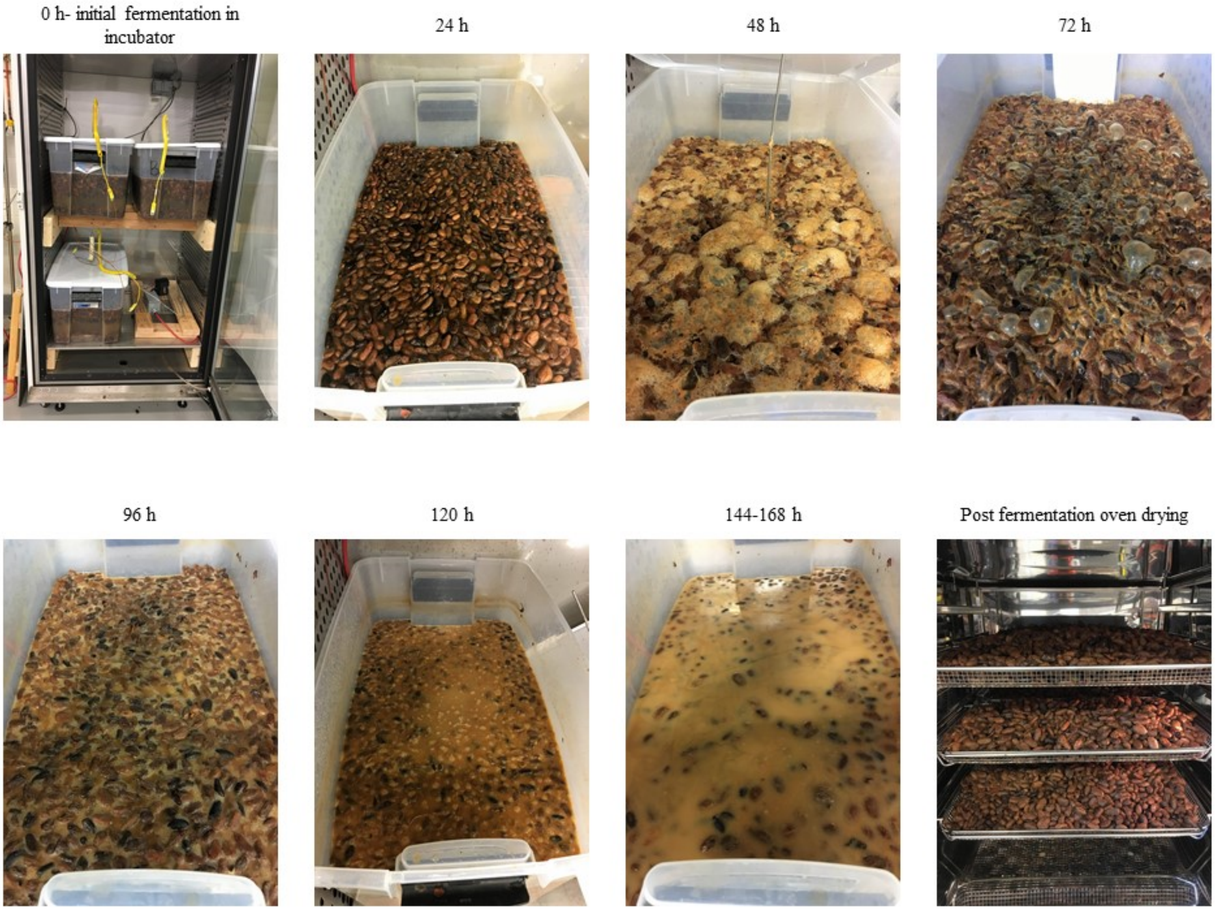

Figure S1. *Progression of one cool fermentation batch from 0 h-168 h, followed by bean oven drying. Fermentation started at 25°C and concluded at 46°C, increasing 3.5°C/24 h.*

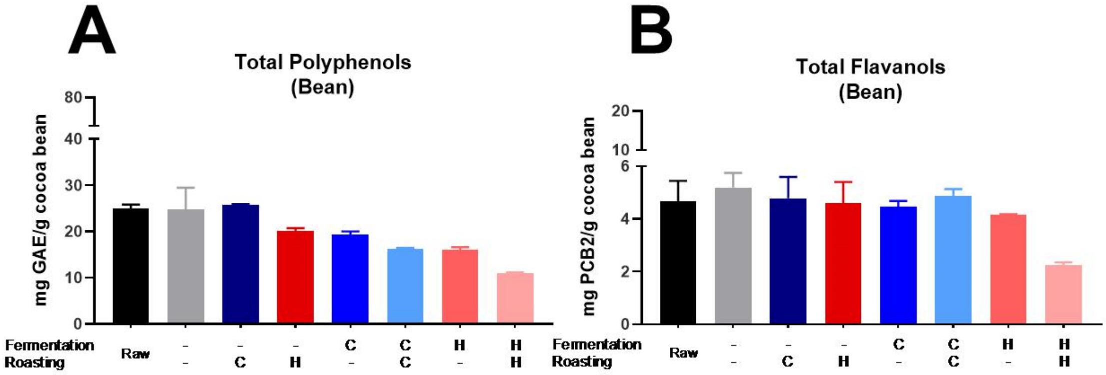

Figure S2. *(A) Total polyphenols, expressed in gallic acid equivalents, and (B) total flavanols, expressed in procyanidin B2 equivalents, of cocoa beans. Raw bean indicates the extract prepared from the cocoa beans as received from the supplier and is different from UF/UR (-/-, which were rehydrated and dried. All values are presented as the mean ± SEM. Statistical analyses were not performed on these data; they are provided for comparison with Figure 2.*

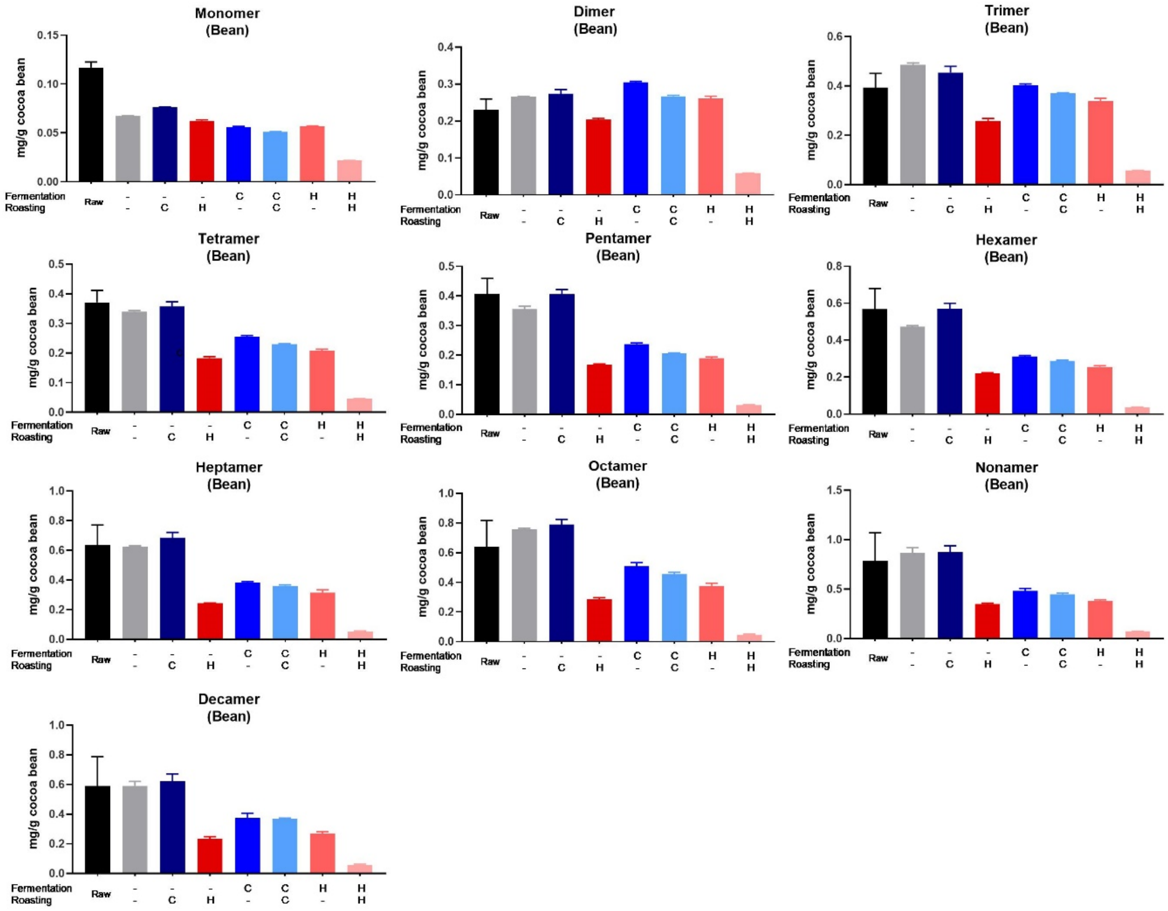

Figure S3. *Levels of individual procyanidin compounds in cocoa beans, as quantified by HILIC UPLC-MS/MS. Raw bean indicates the extract prepared from the cocoa beans as received from the supplier and is different from UF/UR (-/-) as these beans were rehydrated and dried. All values are presented as the mean ± SEM. Statistical analyses were not performed on these data; they are provided for comparison with Figure 3.*

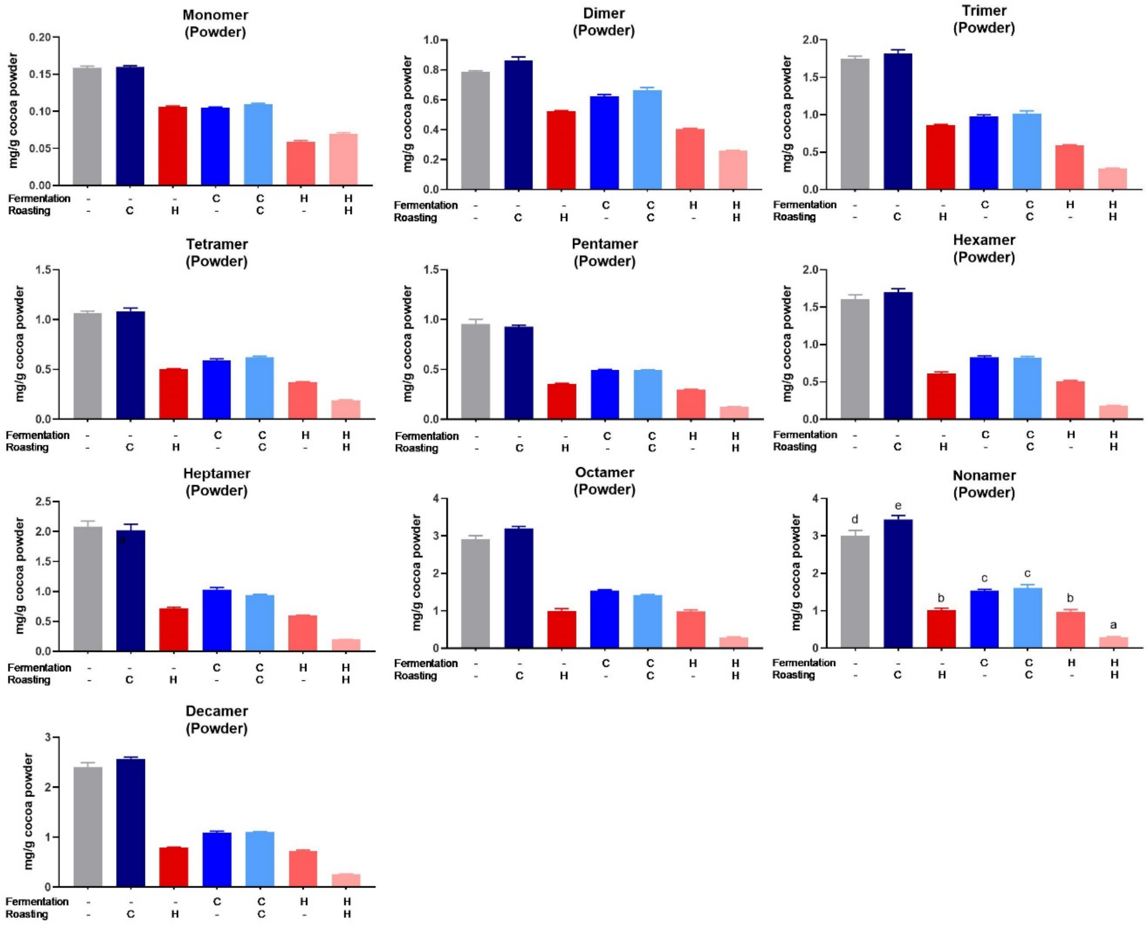

Figure S4. Levels of individual procyanidin compounds in cocoa powders, as quantified by HILIC UPLC-MS/MS. All values are presented as the mean ± SEM. Statistical analyses were not performed on these data; they are provided for comparison with Figure 3.

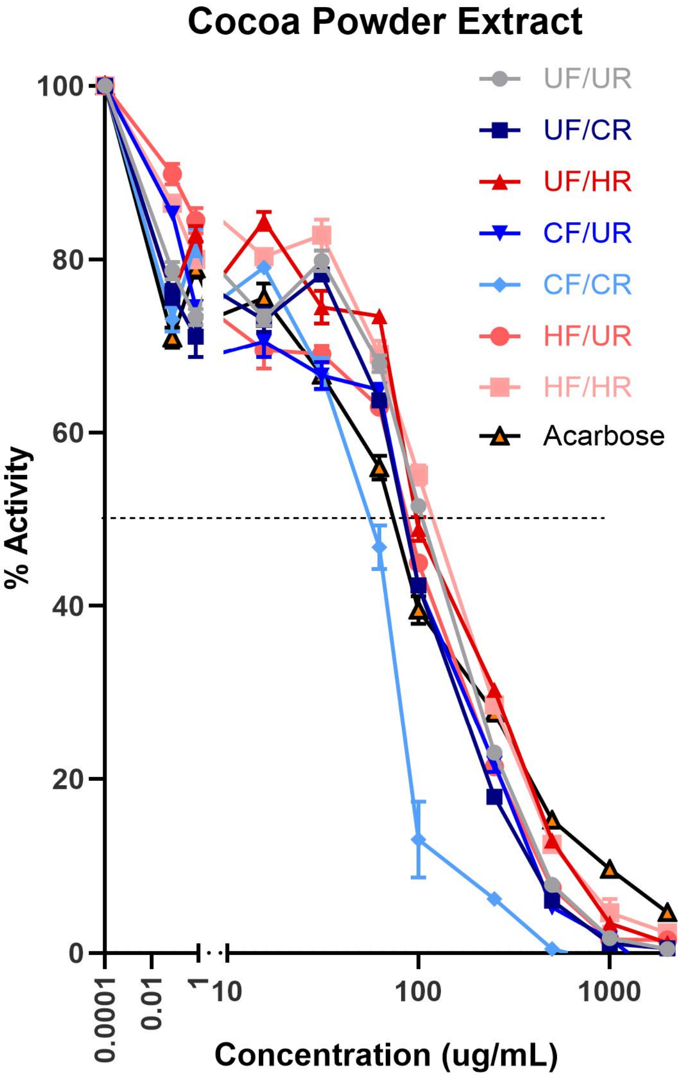

Figure S5. *Dose response curve for α-glucosidase activity (%activity compared to no inhibitor) for cocoa powder extracts. Dotted line represents* IC_{50} *values. Values are presented as mean ± SEM.*
